# Diet and exercise interventions reduce serum asprosin and the corresponding hypothalamic– pituitary–gonad-axis dysfunction in obese men

**DOI:** 10.3389/fphys.2022.896735

**Published:** 2022-09-26

**Authors:** Tingting Yao, Chenglin Song, Yajie Yu, Yang Cheng, Hongyan Lu, Jing Li, Yang Yang, Donghui Tang, Xuejie Yi

**Affiliations:** ^1^ School of Physical Education, Liaoning Normal University, Dalian, Liaoning, China; ^2^ Exercise and Health Research Center/Department of Kinesiology, Shenyang Sport University, Shenyang, Liaoning, China; ^3^ School of Kinesiology, Shanghai University of Sport, Shanghai, China; ^4^ Department of College of P.E and Sport, Beijing Normal University, Beijing, China

**Keywords:** asprosin, hypogonadism, exercise, insulin resistance, hypothalamic-pituitary-testicular axis

## Abstract

**Background:** Asprosin (ASP) is a recently discovered adipocyte factor that participates in glucose metabolism and inflammatory reactions. Recent findings suggest that it may be involved in the regulation of sex hormone secretion in the hypothalamic-pituitary-gonad (HPG) axis, but no studies have been reported in related populations. The purpose of this study was to evaluate the changes in serum ASP levels in healthy men and obese men, as well as before and after exercise weight loss, and to investigate male hypogonadism, insulin resistance, inflammatory response, and relationships induced by ASP and obesity.

**Methods:** Thirty-eight young male volunteers were recruited and divided into a normal group (*n* = 20) and an obese group (*n* = 18) according to their body mass index. Fourteen of the obese men underwent a 14-week exercise and diet intervention (first 8 weeks of aerobic exercise at 60%–70% HR_max_ for 30–50 min/4 days a week). Beginning at week 9, the intensity was increased to 75% HR_max_. Participants in the obese groups maintained a calorie-restricted diet throughout the study period.

**Results:** Serum ASP levels in the obese group were significantly higher than those in the normal group, and serum gonadotropin-releasing hormone (GnRh), luteinizing hormone (LH), and testosterone (T) levels were decreased. After 14 weeks of exercise and diet intervention, serum ASP decreased significantly, the levels of body weight, lean body weight, body fat rate, fasting insulin (FINS), homeostatic model assessment for insulin resistance, TNF-α, IL-6, and IL-1β decreased significantly, and the serum GnRH, LH, and T levels increased significantly. ASP was positively correlated with body weight, body fat percentage, FINS, tumor necrosis factor (TNF)-α, interleukin (IL)-6, and IL-1β and negatively correlated with relative lean body weight and serum LH and T levels.

**Conclusion:** The serum ASP levels were increased in obese men compared with those of normal weight individuals, resulting in a chronic inflammatory reaction, high serum insulin, and HPG axis injury. Fourteen weeks of exercise and diet intervention effectively alleviated this phenomenon. It has been speculated that ASP might regulate male reproductive function by regulating the inflammatory response and insulin sensitivity.

## 1 Introduction

The number of obese people worldwide has almost tripled in the past four decades, and more than 2.1 billion people are currently overweight or obese (Forouzanfar et al., 2017; NCD Risk Factor Collaboration, 2017). Obesity has become a global public health concern. Obesity is an important pathogenic factor of hypogonadism (HG) in men, especially young men (Dhindsa et al., 2010; Shi et al., 2013; Chambers and Richard, 2015). The number of HG cases has also increased dramatically along with the prevalence of obesity. It is estimated that 2.1%–12.8% of adult males are affected by HG, and the prevalence is expected to increase to 6.5 million by 2025 (Molina-Vega et al., 2018). HG can lead to decreased fertility, sexual dysfunction, osteoporosis, and decreased muscle mass in men, and is also associated with an increased risk of type 2 diabetes and cardiovascular disease (Molina-Vega et al., 2018).

HG is a hyposexual disorder characterized by androgen deficiency (Lamm et al., 2016). Multiple studies have shown that serum testosterone is inversely proportional to body mass index (BMI) in overweight and obese individuals. Moderate or greater obesity is often accompanied by HG, and further increases in obesity inhibit the hypothalamic–pituitary–testicular gland (HPG) axis, leading to gonadotrophin hypogonadism (Zitzmann, 2009; Shi et al., 2013; Grossmann, 2018). However, its mechanism of action remains unclear. Studies have shown that obesity impairs HPG axis function through insulin resistance, oxidative stress enhancement, and chronic systemic inflammation, leading to HG (Barbagallo et al., 2021). HG also promotes fat accumulation and insulin resistance, provoking a vicious cycle (Barbagallo et al., 2021; La Vignera et al., 2021). Although complex bidirectional associations between obesity, insulin resistance, chronic inflammation, and HG have been recognized (Saboor Aftab et al., 2013), the specific regulatory mechanisms remain unknown.

Asprosin (ASP) is a new type of hormonal adipokine discovered in 2016 (Romere et al., 2016). It plays an important regulatory role in glucose metabolism in the liver, muscle, and pancreas (Jung et al., 2019; Li et al., 2019). In addition, circulating ASP can cross the blood-brain barrier and directly activate hypothalamic appetite-promoting neurons to stimulate increased appetite (Duerrschmid et al., 2017; Liu et al., 2020). ASP, because of its important role in glycolipid metabolism and appetite control, has been considered a new target for the treatment of metabolic-related diseases, such as obesity.

The changes in ASP among obese people are controversial. Compared with healthy weight individuals, the serum ASP concentration in obese adults is increased (Romere et al., 2016; Wang et al., 2019a; Ugur and Aydin, 2019), while serum ASP concentrations in obese children are significantly decreased (Long et al., 2019; Corica et al., 2021). ASP may have a complex relationship with obesity, which needs to be verified in more clinical studies. Recent animal studies have shown that ASP may be involved in regulating the HPG axis. ASP knockdown in testicular tissue weakens the progressive sperm motility and fertility of mice (Li et al., 2019; Wei et al., 2019). Central ASP infusion in rats can promote sex hormone secretion in the HPG axis, as well as sperm motility and progressive sperm motility (Keskin et al., 2021). However, relevant clinical studies into the association between obesity-induced HG and ASP in the human body have not yet been reported.

Exercise and restricted-calorie diets can reduce body fat and weight and improve HG symptoms secondary to obesity (Giagulli et al., 2019). However, little is known about the effects of long-term physical exercise on ASP concentration in humans (Jahangiri et al., 2021; Kantorowicz et al., 2021). No study has investigated the effect of long-term calorie restriction and aerobic exercise on the serum ASP concentration in obese men and its relationship with the HPG axis. The objectives of this study were to assess ASP levels in obese men and to explore the effects of 14 weeks of aerobic exercise combined with caloric restriction-induced weight loss on serum ASP in obese men and its relationship with HPG axis secretion.

## 2 Materials and methods

### 2.1 Participants

A total of 62 normal and obese male volunteers aged 19–25 years with low physical activity levels were recruited from Shenyang, China. The International Physical Activity Questionnaire (IPAQ) was used to investigate the sedentary time and physical activity of the volunteers, and those with low physical activity were excluded (Qu and Li, 2004). Physical and medical examinations were performed to investigate any history of disease. Individuals with acute cardiovascular and cerebrovascular diseases, liver dysfunction, chronic kidney disease, adverse living habits (tobacco, alcohol, drug use), unstable body weight or body weight changes of more than 5% in the past 2 months, or any restrictions on physical activity confirmed independently by their physician were excluded. Finally, enrolled participants were categorized according to China’s criterion of body mass index (BMI) threshold (Zhou, 2002) into a normal group (18.5 ≤ BMI ≤23.9; *n* = 20) or obesity group (28 ≤ BMI ≤40; *n* = 18). Blood samples were collected for horizontal comparative analysis between the normal and obese groups.

Participants in the obese group underwent a cardiac check-up and a graded stress test, the results of which were used to assess the participants’ ability to exercise at maximum intensity. Exercise- and diet-induced weight loss interventions were performed for 14 weeks. Matched blood samples obtained before (Pre) and after (Post) the 14-week exercise and dietary weight loss interventions were analyzed and compared. Four participants in the obese group dropped out of the study citing a lack of training continuity or personal reasons. Ultimately, 14 men completed the exercise and diet weight loss intervention program.

The study followed the Helsinki Declaration and was approved by the Ethics Committee of the Shenyang Institute of Physical Education. The participants were briefed on the protocol and signed a written informed consent form before the study initiation.

### 2.2 Graded stress test

Graded exercise testing was performed before the intervention phase using a cycle ergometer (Ergoline 100, Ergoline Gmbh, Deutschland) and a respiratory gas metabolism analyzer (AEI MAX-II Metabolic Carts, AEI Technologies Inc., USA). The test protocol was as follows: The participants abstained from vigorous exercise for 48 h before the test, and they received instructions regarding the test method, principle, and precautions on the morning of the test. Participants performed the tests in the fasted state. They were allowed to warm up for 10 min before the start of the formal test. The exercise was initiated at 25 W and was increased by 20 W every 2 min until the participations reached volitional fatigue. Attention was paid to the tightness of the respiratory mask throughout the duration of the test to ensure accuracy of the test results. The maximum heart rate and oxygen uptake observed in this test were considered the maximum heart rate (HR_max_) and maximum oxygen consumption (VO2_max_), respectively.

#### 2.2.1 Exercise intervention protocol

In reference to previous exercise regimens (Atakan et al., 2021; Mercer et al., 2021), during Weeks 1–4, participants performed a 30-min aerobic workout (treadmill or cycle ergometer) 4 days a week at an intensity of 60%–70% of the HR_max_ determined in the graded stress test; from Weeks 5–8 weeks, the intensity was maintained at 60%–70% HR_max_, and the exercise time was gradually increased to 50 min; during Weeks 9–14, the intensity increased to 75%HR_max_. Participants performed 15 min of warm up and relaxation exercises before and after each workout. The participants were instructed by exercise physiologists during all exercise sessions and wore heart rate monitors to ensure their health and to maintain proper exercise intensity.

During the exercise and dietary interventions, participants ate a calorie-restricted diet each week under the guidance of a registered dietician. Body weight dietary intake (55% carbohydrates, 30% fat, and 15% protein) was calculated using the Dietary Reference Intake equation. The daily energy intake was calculated by subtracting 500 kcal from the estimated maintenance-weight dietary intake to achieve a weight loss of approximately 10%.

### 2.3 Body composition determination

A body composition meter (Inbody770, South Korea) was used to test the participants’ height, body mass (BM), BMI, relative lean body mass (RLBM), and body fat percentage (BFP) in the morning fasting state (Mclester et al., 2020). All body composition measurements were obtained by the same examiner.

### 2.4 Blood sample collection

In the control and obese groups, 5 ml of venous blood was drawn from the forearm fossa at fasting state at 7:00 a.m. before and after the study period. After centrifugation at 3,000 rpm for 15 min, the serum was separated into sterile Eppendorf tubes and sealed at −80°C.

### 2.5 Biochemical analysis

The glucose oxidase method was used to determine the fasting blood glucose (FBG) level using a blood glucose detection kit (F006-1-1, Nanjing Jiancheng Bioengineering Institute, Nanjing, China). Fasting insulin (FINS) levels were determined using an enzyme-linked immunosorbent assay (ELISA) using an insulin assay kit (CEA448Hu, Cloud-Clone Corp, Wuhan, China). The homeostatic model assessment for insulin resistance (HOMA-IR) was calculated using the HOMA2 Calculator program based on the FBG and FINS levels (Wallace et al., 2004).

ELISA was used to determine the levels of tumor necrosis factor (TNF)-α, interleukin (IL)-6, interleukin (IL)-1β, interleukin (IL)-10, gonadotropin-releasing hormone (GnRh), luteinizing hormone (LH), and testosterone (T) (rml077385, ml055488, ml058059, ml064299, ml420095, ml026298, and ml064301, respectively; mlbio, China, Shanghai). Each specific operation was strictly performed according to the manufacturer’s instructions.

### 2.6 Serum ASP level determination

Serum ASP levels were determined using an ELISA assay kit (e15190h, ELAAB Science Inc., Wuhan, China) following the manufacturer’s instructions. The assay range of the kit was 3.90–250 ng/ml, and the coefficient of variation (CV) ranged within and between lots as CV<10% and CV<12%, respectively.

### 2.7 Statistical analysis

SPSS21.0 (IBM, Armonk, NY, United States) was used for statistical analysis. The Kolmogorov–Smirnov test was used to evaluate normally distributed variables. Data are expressed as mean ± SEM or as median (Me) and lower and upper quartiles (Q1 and Q3). The comparison between the normal group and the obese group was made using the independent sample T test or Mann–Whitney test, and the comparison between the pre and post interventions of the obese group was made using the paired sample T test or Wilcoxon test. Spearman’s correlation coefficient was used to test whether there was a correlation between ASP and the related indicators. In all statistical analyses, statistical significance was set at *α* = 0.05.

## 3 Results

### 3.1 Comparison of related indexes between normal group and obesity group

**FIGURE 1:**
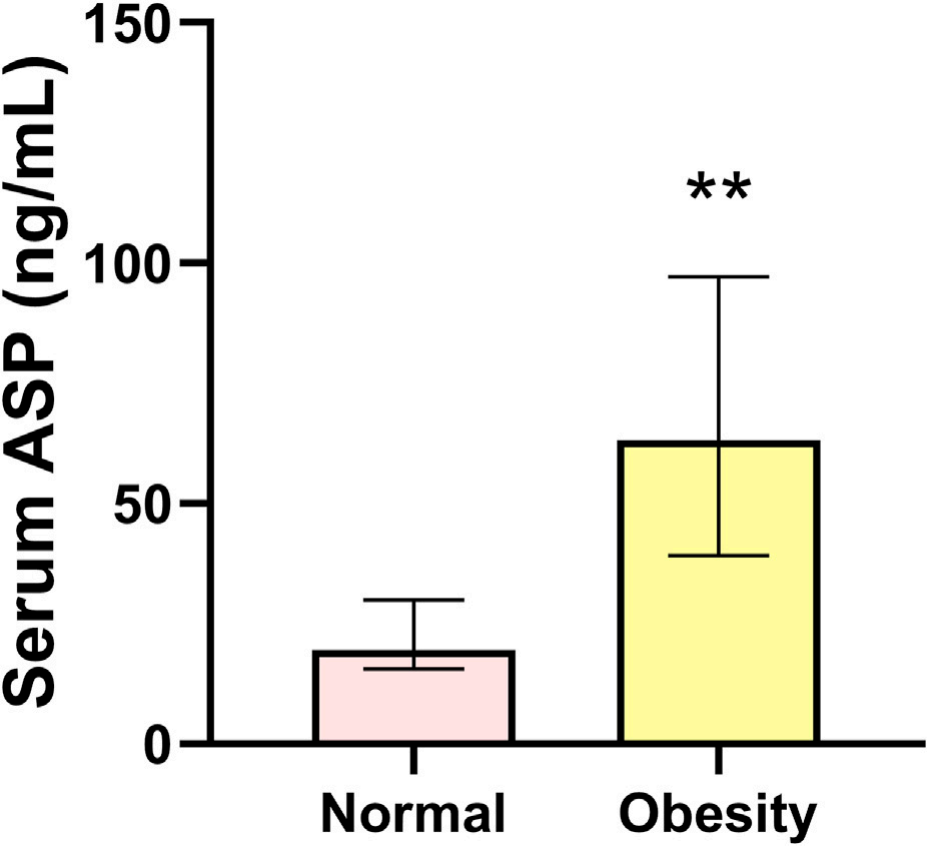
Serum asprosin level in the normal and obesity groups. Data are presented as medians (Me) and quartiles (Q1, Q3); Mann–Whitney test **p < 0.01 for difference from the normal group.

Compared with the normal group, serum ASP levels in the obesity group were significantly increased (*p* < 0.01) (Figure 1). Regarding body composition indices, BM and BFP were significantly increased (*p* < 0.01, *p* < 0.01), while RLBM was significantly decreased (*p* < 0.01) in the obese group compared with those in the control group. For the glucose metabolism-related indicators, FBG was not significantly changed, yet the levels of FINS and HOMA-IR were significantly increased (*p* < 0.01, *p* < 0.01) in the obese group compared with those in the control group. In addition, the levels of the serum inflammatory indices, TNF-α, IL-6, and IL-1β, were significantly increased (all *p* < 0.01), and those of IL-10 were significantly decreased (*p* < 0.01) in the obese group compared with those in the control group. Finally, the serum sex hormone levels, GnRH, LH, and T, were significantly decreased (all *p* < 0.01) in the obese group compared with those in the control group (Table 1).

**TABLE 1:** Comparison of relate variables between normal group and obesity group.

Variable	Normal (*n* = 20)	Obesity (*n* = 14)	*p*-value
BM (kg)	68.77 ± 1.59	108.50 ± 3.05**	<0.001
RLBM	0.86 ± 0.01	0.63 ± 0.01**	<0.001
BFP (%)	14.49 ± 0.95	36.81 ± 1.12**	<0.001
FBG (mmol/L)	4.93 ± 0.15	5.22 ± 0.17	0.207
FINS (μU/mL)	5.21 ± 0.18	12.43 ± 0.41**	<0.001
HOMA-IR	0.98 ± 0.03	1.62 ± 0.05**	<0.001
TNF-α (pg/ml)	17.59 ± 0.70	30.15 ± 1.82**	<0.001
IL-6 (pg/ml)	5.70 ± 0.28	8.22 ± 0.85**	0.006
IL-1β (pg/ml)	1.43 ± 0.06	4.12 ± 0.19**	<0.001
IL-10 (pg/ml)	10.12 ± 0.39	8.14 ± 0.31**	<0.001
GnRH (pg/ml)	29.12 ± 1.39	28.67 ± 1.37**	0.820
LH (IU/L)	8.99 ± 0.13	5.03 + 0.17**	<0.001
T (ng/dl)	572.12 ± 33.03	415.25 ± 34.71**	0.002

Note: Data are shown as mean ± SEM. Independent sample t test **p < 0.01 for difference from Normal group. BM, body mass; RLBM, relative lean body mass; BFP, body fat percentage; FBG, fasting blood glucose; FINS, fasting insulin; HOMA-IR, insulin resistance homeostasis model; TNF-α, tumor necrosis factor α; IL-6, interleukin 6; IL-1β, interleukin 1β; IL-10, interleukin 10; GnRH, Gonadotropin-releasing hormone; LH, luteinizing hormone; T, Testosterone.

### 3.2 Changes of related indexes of obese men after 14 weeks of exercise and diet intervention

**FIGURE 2:**
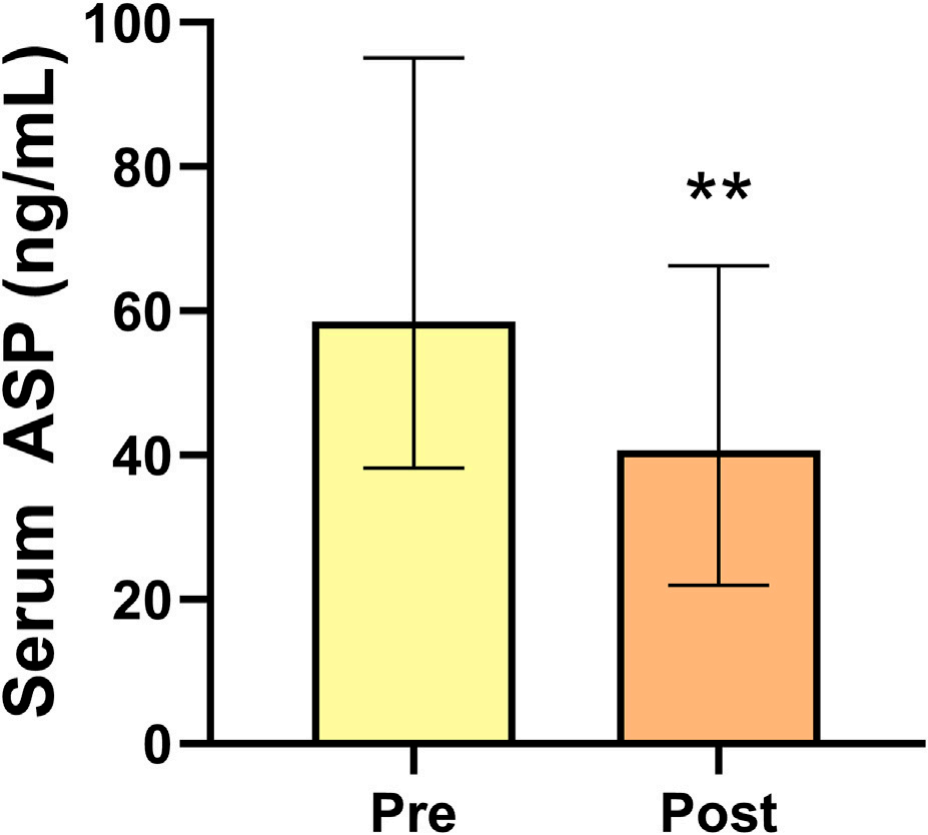
Serum asprosin levels of obese men before and after 14 weeks exercise and dietary intervention. Data are presented as medians (Me) and quartiles (Q1, Q3); Wilcoxon test **p < 0.01 for difference from the Pre group.

Compared with those before intervention, the serum ASP levels of obese men were significantly lower (*p* < 0.01) after 14 weeks of exercise and diet intervention (Figure 2). Both the BM and BFP decreased significantly (*p* < 0.01, *p* < 0.01) in the obese group between pre and post intervention, whereas the RLBM increased significantly (*p* < 0.01). Regarding the indicators of glucose metabolism, FBG levels did not change significantly over the course of the study; however, the levels of FINS and HOMA-IR decreased significantly in the obese group (both *p* < 0.01). Serum inflammation indices and serum TNF-α, IL-6, and IL-1β levels were significantly reduced (*p* < 0.01, *p* < 0.01) at the end of the study compared with those prior to the intervention, while IL-10 showed no significant change. Serum GnRh, LH, and T levels in the obese group significantly increased over the course of the intervention period (*p* < 0.05, *p* < 0.01, *p* < 0.01) (Table 2).

**TABLE 2:** Differences of obesity-related indexes before and after exercise diet intervention.

Variable	Pre (*n* = 14)	Post (*n* = 14)	*p*-value
BM (kg)	109.66 ± 3.79	99.00 ± 3.71**	<0.001
RLBM	0.63 ± 0.13	0.68 ± 0.16**	<0.001
BFP (%)	37.29 ± 1.23	32.57 ± 1.62**	<0.001
FBG (mmol/L)	5.18 ± 0.22	5.04 ± 0.22	0.567
FINS (μU/mL)	12.48 ± 0.53	9.63 ± 0.45**	<0.001
HOMA-IR	1.61 ± 0.74	1.24 ± 0.64**	0.001
TNF-α (pg/ml)	30.14 ± 2.00	24.53 ± 1.37**	0.001
IL-6 (pg/ml)	8.83 ± 0.98	6.93 ± 0.65**	0.007
IL-1β (pg/ml)	3.98 ± 0.21	2.59 ± 0.34**	<0.001
IL-10 (pg/ml)	8.03 ± 0.33	8.41 ± 0.38	0.406
GnRH (pg/ml)	29.41 ± 1.59	31.93 ± 1.12*	0.019
LH (IU/L)	4.96 ± 0.21	7.12 ± 0.17**	<0.001
T (ng/dl)	411.90 ± 42.02	508.37 ± 38.81**	<0.001

Note: Data are shown as mean ± SEM. Paired sample T test **p < 0.01 for difference from the Pre group. BM, body mass; RLBM, relative lean body mass; BFP, body fat percentage; FBG, fasting blood glucose; FINS, fasting insulin; HOMA-IR, insulin resistance homeostasis model; TNF-α, tumor necrosis factor α; IL-6, interleukin 6; IL-1β, interleukin 1β; IL-10, interleukin 10; GnRH, Gonadotropin-releasing hormone; LH, luteinizing hormone; T, Testosterone.

### 3.3 Correlation analysis between serum ASP level and other variables

**FIGURE 3:**
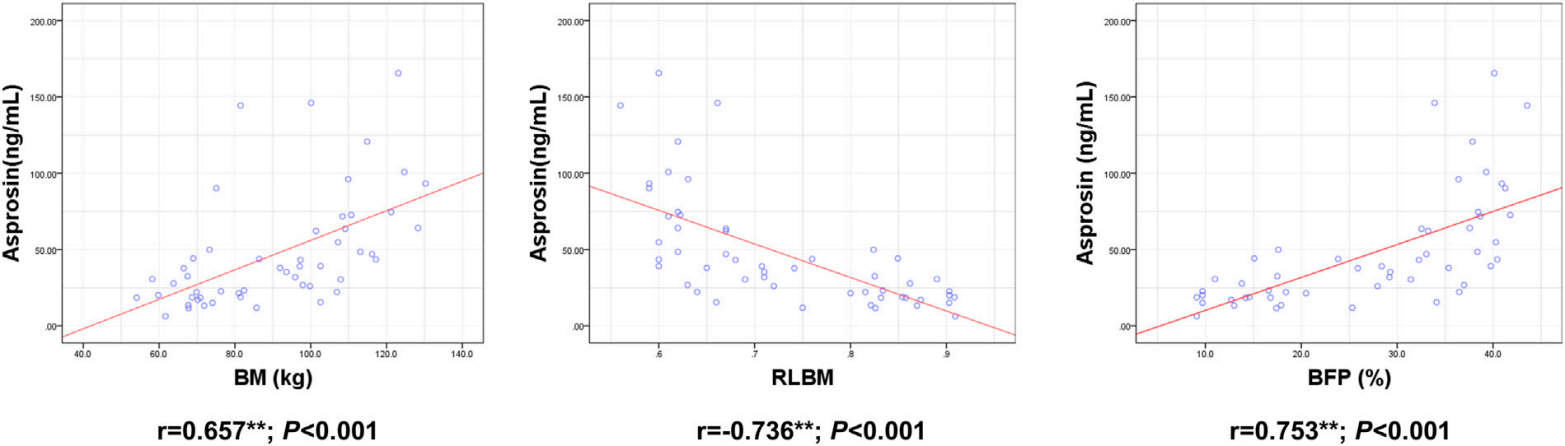
Correlation between Serum asprosin and body composition. r-correlation coefficient, *p* < 0.05—level of statistical significance.

**FIGURE 4:**
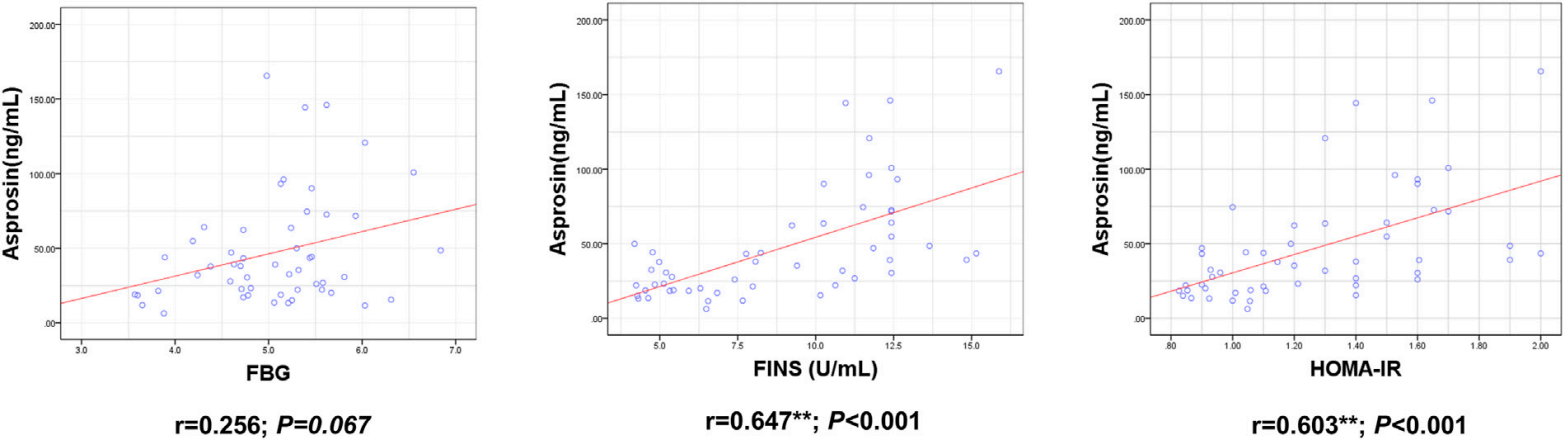
Correlation between Serum asprosin and glucose metabolism related indexes. r-correlation coefficient, *p* < 0.05—level of statistical significance.

**FIGURE 5:**
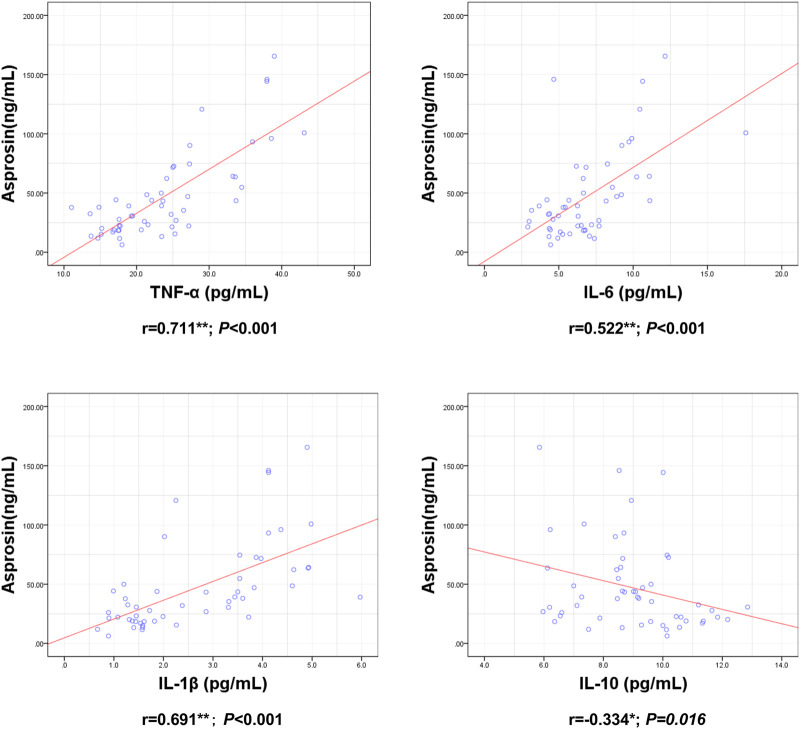
Correlation between Serum asprosin and inflammatory factors. r-correlation coefficient, *p* < 0.05—level of statistical significance.

**FIGURE 6:**
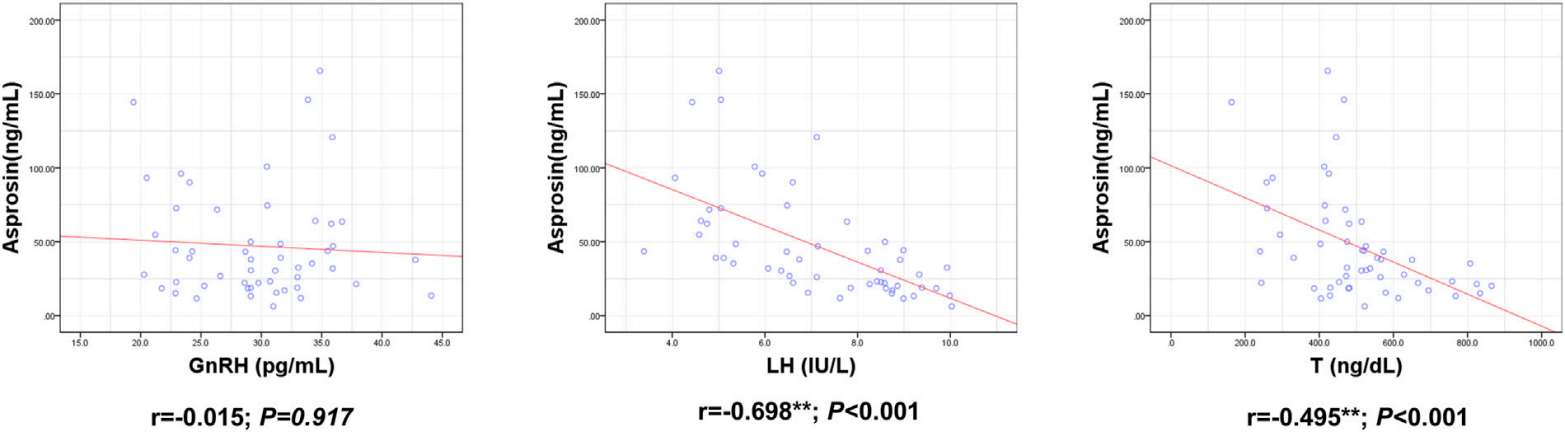
Correlation between Serum asprosin and HPG axis related indexes. r-correlation coefficient, *p* < 0.05—level of statistical significance.

ASP levels were positively correlated with BM, BMI, BFP, FINS, HOMA-IR, TNF-α, IL-6, and IL-1β levels and negatively correlated with RLBM, IL-10, LH, and T levels (Figures 3–6).

## 4 Discussion

Our study shows for the first time that serum ASP in men is closely related to the HPG axis function, and a 14-week exercise- and diet-induced weight loss intervention not only reduced body fat and weight, but also reversed the increase in serum ASP and insulin caused by obesity, and improved the inflammatory reaction and HPG axis disorder.

The role of ASP in obesity has attracted substantial attention. Sunnetci and Wang found that serum ASP levels in obese children were significantly higher than those in normal-weight children (Wang et al., 2019b; Sunnetci Silistre and Hatipogl, 2020). The opposite was found to be true in studies by Long et al. (2019) and Corica et al. (2021). Changes in serum ASP levels in obese populations are controversial, not only among children. The changes in serum ASP levels in obese individuals have also been inconsistent in adult studies. Schumann et al. reported no notable changes in serum ASP levels in obese patients (Wiecek et al., 2018). However, most studies have shown pathological increases in serum ASP levels in adults with obesity (Romere et al., 2016; Wang et al., 2019a; Ugur and Aydin, 2019). Our study also found a significant increase in the serum ASP levels in the obese group. The large individual differences in ASP and the unclear causal relationship and mechanism of obesity suggests a complex relationship between ASP and obesity.

Obesity negatively affects male fertility via various mechanisms (Shi et al., 2013; Grossmann, 2018). Recent research has shown that ASP is involved in the regulation of the reproductive system. The receptor for ASP, Olfr734, is highly expressed in the testis, and knockout of Olfr734 weakens progressive sperm motility and fertilization in mice (Li et al., 2019; Wei et al., 2019). Intraperitoneal injection of ASP into mice increases blood glucose and mRNA levels of ATP and cAMP in sperm, as well as the sperm progressive motility and fertilization rate (Wei et al., 2019). After intracerebroventricular injection of ASP into male Sprague Dawley rats, ASP increased the mRNA and protein expression of GnRH in a dose-dependent manner, as well as the serum LH and T levels; sperm density, sperm progressive motility, and rapid and progressive sperm motility in the testis were also significantly increased (Keskin et al., 2021). Therefore, changes in ASP might be one of the mechanisms by which obesity causes male reproductive problems. Interestingly, our study found that serum ASP was negatively correlated with LH and T. To date, no related literature has been published. However, the fat factor, leptin, can stimulate GnRH release via kisspeptin, which in turn stimulates LH and T secretion (Thompson et al., 2004; Zhang and Gong, 2018). However, in obesity, massive secretion of leptin by adipose tissues triggers hyperleptinemia, which leads to resistance of the HPG-axis to leptin signaling, thus negatively affecting male fertility (Chang et al., 2021; Childs et al., 2021). Therefore, we speculate that in the obesity state, ASP may also excessively activate some negative feedback regulation mechanism, leading to “ASP resistance” and thus inhibiting the HPG axis.

Systemic inflammation and insulin resistance are the mechanisms underlying HPG axis injury in obesity (Barbagallo et al., 2021). Pro-inflammatory factors, such as TNF-α, IL-1β, and IL-6, can inhibit the secretion of hypothalamic GnRH and pituitary LH and reduce T secretion in testicular tissue (Watanobe and Hayakawa, 2003; Kapoor et al., 2008; Bellastella et al., 2019). Insulin stimulates HPG-axis activity at both the central (hypothalamic and pituitary) and peripheral (testicular) levels (Bellastella et al., 2019). However, in obesity, insulin resistance induced by hyperinsulinemia can cause disorders of the HPG-axis (Davidson et al., 2015; Barbagallo et al., 2021). In addition, low testosterone levels in the obese state further aggravate the inflammatory response and insulin resistance, triggering a vicious cycle (Barbagallo et al., 2021; La Vignera et al., 2021). Previous studies have shown that ASP promotes systemic inflammation (Jung et al., 2019) and insulin resistance (Lee et al., 2019). Our study also found that ASP was positively correlated with FINS, HOMA-IR, TNF-α, IL-1β, and IL-6 and negatively correlated with the anti-inflammatory factor IL-10. Therefore, we speculated that ASP may damage the HPG axis by promoting insulin resistance and inflammatory responses.

Exercise and diet control can reduce blood lipids and weight and improve hypogonadotropic hypogonadism and hypotestosterone caused by obesity. Although ASP is a fast-induced appetite hormone (Romere et al., 2016), no effects of caloric restriction on serum ASP levels have been reported. Furthermore, the effect of acute exercise on serum ASP levels is controversial. One-off 20-s bicycle sprints resulted in increased ASP secretion in women, but not in men (Wiecek et al., 2018), while serum ASP in obese individuals showed no significant changes before and after acute exhaustive exercise (Schumann et al., 2017). This indicated that one-off exercise had complex effects on serum ASP due to exercise type, sex, disease state, etc. There are only two reports on serum ASP levels during long-term exercise. The first involved 8 weeks of Nordic walking training at FAT max and revealed reduced blood ASP concentrations in young women with metabolic disorders (Kantorowicz et al., 2021); the second reported that 12 weeks of resistance training also reduced serum ASP levels in sedentary obese men (Jahangiri et al., 2021). Decreases in ASP in both studies were associated with decreases in body fat and obesity indices. Serum ASP concentrations are also significantly reduced after bariatric surgery in obese adults (Wang et al., 2019a). Since ASP is mainly secreted by white fat, it is speculated that the reduction in adipose tissue may be the main reason for the improvement of high serum ASP in obesity. To test this hypothesis, we selected aerobic exercise combined with calorie restriction as an intervention modality with good lipid reduction effects. Our results showed that 14 weeks of aerobic exercise combined with dietary intervention significantly improved obesity and reduced serum ASP levels. Serum ASP was positively correlated with body weight and body fat rate, and negatively correlated with relative lean body weight. These results suggest that long-term exercise and dietary intervention can improve the high serum ASP levels of obese people by reducing body fat. Our study also showed that 14 weeks of aerobic exercise combined with dietary intervention resulted in a reduction in serum ASP concentrations, while improving insulin resistance, inflammatory response, and promoting HPG axis hormone secretion. This further verified that ASP might damage the HPG axis by promoting insulin resistance and inflammatory responses.

At present, there are few reports concerning ASP regulation of the reproductive axis, and those of serum ASP levels in the normal and obese states are controversial. This study shows that the high serum ASP level in obese people is negatively correlated with the HPG axis function. Referring to leptin results, it is speculated that ASP resistance may exist in the obese state, but unfortunately, we lack substantial evidence to confirm this point in this study. Further studies of human or animal experiments are needed to verify ASP resistance in the obese state.

Although our research shows that ASP is involved in the influence of obesity and exercise on HPG axis, the influence of obesity on ASP differs between adults and adolescents. Further research to determine the relationship between ASP and HPG axis function in obese adolescents could elucidate the relationship between ASP and the male reproductive system. In addition, the influence of ASP on the female reproductive system has not yet been clarified. Further research is needed in this field. Nevertheless, we believe that this study linking serum ASP and HPG axis function provides an excellent foundation for these future studies and may provide a basis for new protocols in dietary and exercise interventions.

## Data Availability

The raw data supporting the conclusion of this article will be made available by the authors, without undue reservation.

## Abbreviations

HG, hypogonadism; HPG, hypothalamic–pituitary–testicular gland; BMI, body mass index; RLBM, relative lean body mass; FBG, fasting blood glucose; BFP, body fat percentage; FINS, Fasting insulin; ELISA, enzyme-linked immunosorbent assay; TNF-α, tumor necrosis factor; HOMA-IR, homeostatic model assessment for insulin resistance; IL, interleukin; GnRh, gonadotropin-releasing hormone; LH, luteinizing hormone; T, testosterone; CV, coefficient of variation.
